# The Outlook of Medicine and Surgery. No. 2

**Published:** 1883-07

**Authors:** J. Mc. F. Gaston

**Affiliations:** Campinas, S. Paulo, Brazil


ATLANTA

### MEDICAL REGISTER.

VOL. II.] JULY, 1883. [No. 10

### Original.

### THE OUTLOOK OF MEDICINE AND SURGERY.

Number 2.

BY J. MC. F. GASTON, M.D., CAMPINAS, S. PAULO, BRAZIL.

In the practical duties of the medical profession there is much to interfere with scientific investigation, yet the two should progress harmoniously and in mutual dependence upon each other. There is a proneness in the common apprehension of mankind generally to consider men of science as theorists without capacity for undertaking the details of treating diseases, and while perhaps there is some foundation for such an impression, it is incumbent upon those who are engaged in the several branches of the practice of medicine, surgery and gynæcology to a disabuse the public mind of such a view of the profession, by bringing into their various fields of service the highest order of attainments that may be practicable when entering upon their work, and by all the application and diligence in study that may be compatible with the demands of their patients upon their time and attention. When it is said that a practitioner is too busy in treating his cases to inform himself of the progress made in the wide domain of experience by others, it is a fair inference that he wilfully declines to receive instruction, and that his egotism induces him to suppose that all outside of his individual observation is unimportant for securing good results in the application of means of relief in his sphere of operations. This radical error of the vontinist is exposed by a comparison of his mode of accomplishing results with the course pursued by a colleague who keeps up with the advancement of knowledge in the medical world, and who exercises sound judgment in selecting the most efficient measures of treatment for diseases. That some men are endowed by nature with more practical tact than others, and even with less study, jump at conclusions as to the diagnoses and treatment of cases more in accordance with the true condition of things, does not militate in any way against the broad principle of enlightenment adding to efficiency in practice. Every practitioner in any professional field requires a base work of an elementary kind in the first place, upon which a solid superstructure may be built up by gradual accessions of materials derived from all the sources of knowledge available to him. Not only should he pursue a systematic cause of study in the standard text books, and reap instruction from the masters in the various branches taught in medical schools, but he should have a well stocked library of sterling works for consultation upon the various disorders that come under his observation from day to day. It might be requisite for a proper comprehension of the work done by specialists to consult half a dozen authors upon a given disease, and thus get the cream of experience so as to be utilized in making up the map of knowledge as a guide to proceeding in a critical case. No medical man can of course undertake to read all the books he needs to have upon his shelves, yet he may as occasion requires cull from each that which serves his purpose in practice. The responsibility of a practitioner to accomplish all that the lights of science enable him to do for his patient, renders it imperative to keep abreast of the progress in all departments of hygiene, so as to avert the development of preventable epidemics, and guard against the access of disorders from special local causes of disease. With all the precautions practicable against vitiation of the atmosphere, the invisible and inappreciable decompositions of the elements that compass the earth will continue to exert a prejudicial effect upon health, and when the influence of these morbific agents is reduced to the minimum, it will still be found that physicians have an ample field of service in correcting the disturbances of the animal economy in men, women, and children. To the honor of our noble corps of hygienists, measures have been adopted under the patronage of the U. S. government, which tend to the amelioration of those sections which have been most scourged with yellow fever. While there are some who do not accept the doctrine of spontaneous development of this disease in our Southern ports, looking to foreign importation for its origin, the data suffice to attribute it in some instances at least to local causes; and those charged with the health regulation of such places have instituted vigorous measures for drainage and cleanliness. As to the source of yellow fever much uncertainty exists, and whether it is in any form identical with that of ordinary malarial fevers is not satisfactorily determined ; yet that a certain degree of heat calculated to produce decomposition in the local deposits is an essential to its production is conceded by all.

Certain conditions of joint residence for some considerable period on the part of a population in a given locality, leading to the accumulation of deposits of fecal evacuations and the offal from kitchens, etc., are the concomitants, if not the requisites, for the propagation of yellow fever; so that we should look to this combination as the most probable source of the exhalations that contaminate the atmosphere. In the year 1876 I had an opportunity of observing some cases of a malignant fever, with all the characteristics of yellow fever, in the city of Campinas, in Brazil, which seemed to have their origin from a covered drain, with openings in the rear of several houses, that served as the receptacle of the waste water from the kitchens. During the rainy season the water flowed freely from the gutters of their shed and carried away the accumulations, but at the time of the eruption of this fever no rain had fallen for more than a month, and with the moisture and warmth decomposition was set up in these materials, so as to affect the adjacent air in a manner to produce this disease.

It is rare that circumstances afford such tangible proof of the origin of yellow fever as were here presented, and the facts should serve as an illustration of the manner in which local causes may be instrumental in producing this disease. Let those more competent than the writer turn this isolated case to account, in elucidation of the predisposing decompositions that lead to the most serious vitiation of the atmosphere.

When preconceived therein, and set aside by accurate observations of facts, and the patient investigation of cause and effect, in the development of epidemics, we may expect the dissemination of more correct views of hygiene and the application of more efficient therapeutic measures. The discussions as to hygiene indicate such preoccupation in favor of the special schemes of the parties as to preclude a just and fair appreciation of the data presented, and this subject has unfortunately become a hobby horse for some to ride into popular favor without any reference to the true merits of the case. The plans for disinfecting vessels and cargoes are in some instances really farcical, and remind one of the brushing dust from the feet or shedding the sandalsbefore entering a heathen temple for the worship of imaginary deities. But in the multitude of counsellors there is wisdom, and some good result must ensue upon the principle of exclusion, so that in the end a trustworthy process should be adopted. Agitation must eliminate error and eventually establish the truth in this, as in other issues.

The vast proportions of the field for hygeiene and prophylactic operations may well engage the best talent of science; and in this age of specialties it is destined to be elevated into a special department of study for philanthropic aspirants to distinction. He who cures the ills that flesh is heir to, deserves undoubtedly grateful recognition from those who suffer, but he who stays the access of disease should receive the high and n oble word of praise from mankind generally in their "well done, good and faithful servant."

If it were practicable for physicians to adopt the plan which an Eastern ruler resorted to with his medical attendant for the compensation of his time while free from disease and stopping his salary whenever sick, it would prove a great incentive to the perfection of hygiene. Not that medical men are indisposed to promote health regulations among the people, yet there is a deep seated element of self-interest that operates upon man in all the relations of life, and when properly directed is really the final cause of our best actions, as the promotion of the welfare of others is the source of greatest enjoyment to an individual. In this point of view it should appeal to our honorable sentiments toward our fellow beings, and to our sense of self respect, to advance the well-being of mankind by eradicating, so far as possible, the seeds of disease from all sources of contamination. Should, however, the pecuniary interest of the medical man be promoted by keeping his clients well, instead of receiving his pay for attendance upon them when sick, it is evident that it would stimulate him to greater zeal in the investigation of the sources of disorders of every kind and to more efficiency in the promotion of hygiene regulations in all the phases of society. The extensive ramifications of the details which tend to the arrest of physical disorders amongst the different classes of men, women and children opens up a vast field of study, which has thus far received but little consideration even from those whose attention has been directed to general hygiene; and the people need to be educated up to the point which may fit them for profiting by instruction in these matters.

Littre defines hygiene as that part of medicine which treats of the rules according to which a choice is made of the means proper for maintaining the normal action of the organs at different ages, in different constitutions, in the different conditions of life, and in the different avocations and professions. It comprehends really the employment of things, whether outside of the individual or emanating from the person, and directs their use as may be required for the preservation of life and health.

The most general division of hygiene is into those agencies which operate upon the mental powers and those which affect the physical development, and the means adopted may be public or private, according to the source of the regulations.

The proceedings for effecting hygiene modifications in the municipal, commercial, naval and military departments differ materially from those indicated for social and domestic security, while the precautions for individual protection fall within the domain of personal consideration—*sana mens in corpore sano.*

Hygenie treats of the preservation of health in all the relations of life, and "embraces a knowledge of healthy man, both in society and individually, as well as of the objects used and employed by him, with their influence on his constitution and organs." Physiological perfection in the human organization is the true expression of the ultimate success of hygiene in all its bearings upon the human race, and is the grandest achievement to be aimed at by man.

Next in importance to the relations of the physician to his patients is his attitude towards his colleagues and others who claim to cure disease.

Some hightoned members of the medical profession have assumed the existence of a tacit code of honor among well educated regular practitioners, that should suffice to regulate all their dealings with each other without any reference to prescribed rule of conduct. Yet it is incumbent upon us to recognize the fact that laws are enacted among enlightened people to provide against the departures from the standard of propriety in their reciprocal associations, and it cannot be ignored that not a few enter into competition for practice without any regard for the dignity of the profession, who are prone to seek their personal interests independent of the rights of others. For the control of such associates, and for the guidance of all the members of the medical fraternity, certain stipulations have been found necessary to define their bearing towards one another, which serve as the chart and compass by which to steer our course safely through the sea of strife before us. As the great augmentation of population has lead to increase in the number of medical men and the competition for favor among the people has become more active, it is now requisite to adopt more stringent rules for the regulation of their deportment, so that each shall not simply understand his sphere of duty, but shall be held responsible for transgressing the prescribed limits. Instead of generalizing medical ethetics the circumstances which surround the profession at present, call for regulations in detail to meet the exigencies of daily issues between the worthy and the unworthy competitor for practice in the United States.

The recent agitation growing out of the action of a small faction in New York, having withdrawn the barriers to consultation with irregular practitioners of medicine, indicates a settled policy on the part of the medical profession generally to maintain the former status of ethics in this respect. Though some reputable members of our profession have for the present compromised their dignity by yielding to a mistaken notion of humanity in acceding to the sly trick of homeœpathists for recognition, it is not likely that their example will be contagious. The fruits of this experiment will satisfy all who are actuated by proper principles that such consultations can not be attended with any practical benefit to either patient or physician, as there can be no common ground for a conference as to the means of cure: If the regular physician responds to the call for consultation with a homeœpathist, of course he must accept any demand made upon him for a homeœpathist to be called into consultation with him in the treatment of his own cases, and allow full weight to his grave suggestion for the use of his infinitesimal doses of medicine upon the principle of *smiclia similibus curantur.*

If self interest has been at the bottom of this perversion of ideas, we must expect to find all these would-be philanthropists, acquiescing in this legitimate result, rather than abandon their charge when the man of globules makes his appearance, and the true sentiment of honorable trust will be frittered away in the degrading farce of this hybrid consultation. Should there however remain some sense of integrity on the part of the advocates of this cunning scheme the law will soon become a dead letter on their statute books.

I would commend to those who wish to be hail fellow well met with homeœpathists the example of a regular graduate of the medical school of this de Janeiso, who has been engaged in the joint practice of the opposing doctrines of Hippocrates and of Hahnemann in the city of Campinas, during my residence there, and published in the *Gazeta de Campinas* of the 22d of April, 1883, the following card:

"Reperotorio etiologico de indicacœs homœopaticas:

"I have just read with attention the work of the intelligent practitioner, Senr. Yoao Baptista Morato do Canto.

"To the friends of this system of medicine, I advise the reading of this book, which can be very useful to them in the absence of a physician and of other resources.

"The work is one of the best that I know of, by the clearness and method observed by its author, who merits the praise and support of all the friends of humanity; since, without pecuniary means, he has rendered a good service to the public, by employing his precious time in a useful work. DR CASSIANO B. DE NORONGA GONZAGA."

That a proper appreciation may be made of this proceeding it is requisite to state that the author of this homœopathic production is a man of limited mental capacity and little education who has been beating around from pillow to post as an irregular practitioner of this humbuggery for a number of years, and his tract is as good for nothing as can be gotten up anywhere.

At the close of a hundred and forty-two pages of this nonsense, the pamphlet closes with an injunction which is strictly in keeping with the tenor of its previous nothingness, and yet gives a correct impression of the true creed of the homœopathist, and of which I append a translation, that others may appreciate the frankness of this blind leader of the blind.

"If the patient insists upon taking more doses without reason, go on giving him pure water in clean vials with fancy labels.

"If he wishes daily visits, though not necessary, go on making them, it being certain that if they are not made, you will be supplanted by another; and will thus lose fame which is so desirable, and money that is so requisite

YOÃO BAPTISTA MORATO DO CANTO,
Professor de Homeœpathia."

It is not supposed that many of those who are defending consultation with homeœpathists are ready to embrace the practice of this time serving substitute for medical treatment or to excuse such trifling with the responsibilities of the physician, yet in so far as the regulation provides for meeting with these practitioners and discussing the means to be used in the cure of disease, they become recognized as members of the medical profession, in a way that imposes upon the physician so consulting with them the obligation to give weight and authority to their views and opinions as to the treatment of the cases.

There is no sort of embarassment in taking charge of a case which had been under homeœpathic care and thus all the claims of humanity may be met.

In regard to those outsiders who claim the sanction o law, and the pretenders who explain the credulity of the people without legal authority, the judicious exercise of masterly inactivity recommends itself by every consideration of self-respect. If regular physicians, with a proper sense of dignity in their bearing towards one another, will ignore completely all irregular practitioners of whatever name or rank, leaving them and their dupes to reap the fruits of their folly, it will prove the most effective rebuke that the medical profession can administer to this class. As a mainspring of action, all should seek to advance the standard of worth among the members of the craft to which they belong, and take no step which may be detrimental to the brotherhood, thus giving mutual support to one another under all the trials and difficulties of practice. The delicate and responsible attitude of a consulting physician exacts the greatest circumspection as to the attending physician, as the family and friends of the patient are prone to seize upon a word or a look of one who is sought as a counsellor that may reflect unjustly upon him who has been in charge of the case. Never, under any circumstances, should the conference be held in the presence of the sick person or of other persons, either belonging to the family or persons casually in the house The freedom from restraint necessary for a proper presentation of the views of either physician, can only be enjoyed by the exclusion of those not identified with the past and future management of the case, and any new feature of treatment that may be agreed upon, should be carried out upon their joint responsibility. My personal experience of consulting in the sick room with eager listeners, during my residence in Brazil, has confirmed me in the conviction of the disadvantages growing out of such a course, and in returning to take my place among my professional colleages I may be allowed to raise my voice in unmistakable tones against all and any participations by others in the deliberations of physicians as to the manner of treatment to be adopted for a patient under their charge. The temptation on either side to be influenced by the bystanders in the expression of their views, should be effectually blocked by closing the door against all intruders, and apart from the unnecessary examination of the sick and the history of the past treatment nothing should transpire as to their respective opinions. No talking for buncombe is admissible in a medical discussion, and the only effective bar to it is that all consultations be held in secret.

Whether a consultation has been requested by the attending physician, or has been suggested by the friends of the patients does not effect the case of exclusion, and even if there should be no divergence between the consultants, the same caution should be observed. as a safeguard to all concerned, upon the general principles.

It may seem a work of supererogation to dwell thus upon this point, but I am profoundly impressed with the benefits to be derived from more frequent consultations in critical cases, and every obstacle should be removed to seeking such a conference whenever desirable to either party. The strict adherence to the rule indicated must relieve this of all embarrassment.

Among the wide-spread applications in medical and surgical practice at the present epoch nothing is more worthy of careful consideration than the use of anæsthetics. The important results attained within the comparatively brief period of the extensive resort to this class of agents, is perhaps the most remarkable advance made for the relief of suffering humanity. It strikes the observor now as surprising that many surgical operations should have been undertaken forty years ago without having anything that gave the kind of immunity from pain that has been afforded by sulphuric ether, chloroform and bromide of ethyl in such a number of cases in later years. Not only in surgical cases, but in medical treatment, these pain-killers have gained grand victories in the hands of practitioners throughout the civilized world, and the medical profession have recourse to them under such varied conditions and in such a vast number of cases that they seem really indispensable in practice. If we should be deprived for a time of anæsthetics, such disturbances of of the established order of proceeding would ensue as to upset all our plans for efficient treatment of diseases; and we could dispense with any other class of remedies with less derangement of the therapeutic programme than by the loss of their inhalation. While some have employed one to the exclusion of others, all concur in the necessity for an anæsthetic, and there are few physicians or surgeons who do not resort daily to one or another article of this class. Though the statistics show that fatal results occur sometimes from imprudence in the administration of each agent, and even from nitrous oxyde, yet very few who are engaged largely in surgical practice have been deterred by this rare occurrence from using one or another as occasion required.

The latest candidate for public favor had well nigh been abandoned from a rather large percentage of untoward results early after its first introduction. But a new champion of the bromide of ethyl has brought it again to the front, under more favorable auspices, and it now promises to be a safe and efficient instrument in the hands of those who require a prompt and temporary insensibility, during speedy operations upon the eye and other organs. The nitrite of ethyl has also been found trustworthy in similar cases by other observers, but I confess that the few cases in which an opportunity has been afforded for its employment, have not given me much encouragement for future experiments with it. The standby for the larger proportion of surgeons has been chloroform, and having had a considerable experience with this faithful friend, I am not disposed to set it aside for any other anæsthetic. Early after it was first brought to the notice of the profession I employed it in all important surgical operations, and during the war of subjugation numerous occasions were presented for its application in my own sphere of duty and in that of others under my observation as chief surgeon of division in the army. Since that period, chloroform has been my sheet anchor in all operations of moment, and never yet has it been my misfortune to witness a fatal result connected with its inhalation. I have read reports of death from chloroform, as in like manner from sulphuric ether, but shall always believe that with due precaution in the mode of administering these articles a fatal issue will be averted. There are very few agents which are patent for good that may not be so abused as to produce evil; and even if it were patent that a certain limited number must die from the inhalation of chloroform and ether, we would still be warranted by the vast proportion of the salutory effects, in resorting habitually to the use of these anæsthetics. Does anyone think for a moment that because of the terrible disasters growing out of carelessness in the employment of steam as a propeller we should cease to use it as an effective means of locomotion. Or coming here to our own domain, that on account of a fatal accident now and then from the use of the surgeon's knife, that we should not resort to cutting instruments for performing important operations upon the human body. Let us then act upon the same common sense view of our surroundings as to anæsthetics, and continue with prudent forethought to give our patients the benefit of chloroform, ether, ethyl, nitrous oxide, or even *hurried respiration*, as occasion may require. Death occurred more frequently under the old regime upon the operating table than it has done under the benign influence of God's best gift to the surgeon in the shape of anæsthetics, and it should not be as carecrow to operators.

The origin of trouble in most cases is from asphyxia by the want of oxygenation of the blood through the lungs. All operators must have observed that when a subject is profoundly under the influence of chloroform, that the arterial blood takes on the dark hue of venous blood, thus showing that it has not undergone the change which ordinary contact with the air in respiration induces. The atmosphere has been shut off by the blood, so that eventually the action of the lungs cease from the lack of nerve power through the centres, and artifical respiration becomes necessary to avert serious results. It has frequently occurred under my observation that a temporary suspension of the action of the lungs has been promptly relieved by artificial respiration, and the use of the chloroform has been continued subsequently without further trouble. A sharp slap or two over the pectoral muscles, or sudden pressure over the points of the ribs, will suffice generally to excite the lungs when there is slight interuption; but if this fails, the patient should be rolled from the back to the side inclining the chest downward by jerks, and repeated, until respiration is again established. Should the suspension of respiration be prolonged, the application of electricity is indicated; placing one pole on the back of the neck and the other carried from point to point over the chest. Douches of cold water over the face and chest also, should be resorted to, for arousing the dormant nerve centres by their revulsive action.

Though the prolonged inhalation of chloroform may be attended with some risk, it may be safely employed for the production of its first temporary insensibility to pain, which admits of operations that require but little time, as lancing tumors dividing strictures of the urethra, extracting teeth, etc. This transient effect occurs while the patient still has some degree of consciousness, and if educated in regard to the phenomena which characterises it, the person may indicate the moment for proceeding with the operation without suffering pain. When the buzzing in the ears and the sense of expansion in the brain becomes well developed under the inhalation of chloroform, brief operations, otherwise of a painful character, may be executed, while still aware of every step, and yet without any discomfort to the individual. All who have been accustomed to note the influence of chloroform at its different stages must have become aware of this early insensibility of a transient nature, which having passed, it becomes necessary to produce its complete narcotising effect for the performance of any protracted operation. It is at the moment of this temporary insensibility that I have repeatedly executed quick steps in surgery, and it has been my personal insensibility to the pain of extracting several teeth under this primary anæsthetic influence which assures me that while still aware of what is done there is really no suffering. I have taken a hankerchief charged with chloroform in my own hand and inhaled it until the sound as of many waters filled my ears, when the word has been given to proceed, and the dreaded extraction was effected without any discomfort whatever. Try it, if you would believe it.

The most notable effect of chloroform is the influence propagated to the nervous system through its external application to the peripheral distribution of the nerves in the cutanous tissue. The experiments of Brown-Sequard demonstrate a relation between the capillaries and nerve fibres of certain parts with other regions of the body, by which an inhibitory action is developed by the local application of this agent; and we may anticipate the transmission of the most important modifications to the vital functions by this discovery. I have awaited with anxiety the publication of the details of recent experiments by this scientific physiologist, and if I mistake not still more astounding revelation will be made ere long, than those already reported in regard to his experiments upon the inferior animals, illustrating "arrest of the exchanges."

The future developments elucidating obscure function of the nerve-centres and the reciprocal action and reactions between the peripheral termination of the nerves and the internal organs are destined to be among the most important advances that have been gained for physiology in the last half of the nineteenth century. Much that has been mere conjecture and hypothesis will give place to correct theory based upon a proper interpretation of facts. The investigations of painstaking experimentalists are bringing out the true explanation of phenomena that have heretofore been accunted for erroneously, and the mutual dependencies of different parts which have been vaguely attributed to sympathy, must have a rational and philosophic solution in a due comprehension of cause and effect.

All are aware of the striking and speedy influence of the outward application of chloroform in relieving what is commonly styled nephritic colic, spasm of the gall duct, and neuralgic pain of all sorts, but the key to all medication through the circulation of the nerves and capillaries with the nerve-centres opens a new field of investigation for physiologists. The clinical observations made in the light of increasing knowledge as to the real interpretation of facts, will have a new signifieance for the pathologist. The systematic results of the adaptation of means to the end in view will lead to vast improvement in therapeutics by the practitioner. Thus it may be confidently anticipated that many disorders of the nervous system whose etiology is inadequately comprehended or assigned to general derangement of the nerve centres, will be traced to special disturbance of certain functions, by the correction of which the whole train of consequences will be renewed. The dark shadow which looms up before the investigation of the source of hysteria, the terra incognita of etiology, the glimmering light cast upon neuralgia, and a general uncertainty as the condition ordinarily styled nervousness, appealed to the critical observer for mature consideration, in view of the recent developments of science. That many impressions are primarily upon the peripheral distribution of the nerves, and thence propagated to the alimentary canal,thus radiating the supplies to the various organs and tissues is evident to every close observer of facts, and by reciprocal action the deranged functions induce disturbance generally.

